# *Synthetic* TILs: Engineered Tumor-Infiltrating Lymphocytes With Improved Therapeutic Potential

**DOI:** 10.3389/fonc.2020.593848

**Published:** 2021-02-16

**Authors:** Anaïs Jiménez-Reinoso, Daniel Nehme-Álvarez, Carmen Domínguez-Alonso, Luis Álvarez-Vallina

**Affiliations:** ^1^ Cancer Immunotherapy Unit (UNICA), Department of Immunology, Hospital Universitario 12 de Octubre, Madrid, Spain; ^2^ Immuno-Oncology and Immunotherapy Group, Instituto de Investigación Sanitaria 12 de Octubre (imas12), Madrid, Spain

**Keywords:** cancer immunotherapy, adoptive cell therapy, tumor-infiltrating lymphocytes, genetically engineered TILs, synthetic TILs

## Abstract

Immunotherapy has emerged as an effective and life-changing approach for several types of cancers, both liquid and solid tumors. In combination with traditional treatments such as radiotherapy and/or chemotherapy, immune checkpoints inhibitors have improved prognosis and overall survival of patients with advanced melanoma and many other cancers. Among adoptive cell therapies (ACT), while chimeric antigen receptor T cell therapies have demonstrated remarkable efficacy in some hematologic malignancies, such as B cell leukemias, their success in solid tumors remains scarce due to the characteristics of the tumor microenvironment. On the other hand, ACT using tumor-infiltrating lymphocytes (TILs) is arguably the most effective treatment for metastatic melanoma patients, but even if their isolation has been achieved in epithelial tumors, their success beyond melanoma remains limited. Here, we review several aspects impacting TIL- and gene-modified “*synthetic*” TIL-based therapies and discuss future challenges that must be addressed with these approaches.

## Introduction

### Adoptive Cell Therapy in Cancer

The spectrum of cancer treatments has been increasing in recent years with the incorporation of different immunotherapy strategies that take advantage of the effectiveness and potential of the immune system to fight cancer cells. Cancer immunotherapy has been named 2013 *“Breakthrough of the year*” by the prestigious Science journal (1). Different immunotherapy approaches are currently under development aiming to improve outcomes for cancer patients, such as immune checkpoint inhibitors [CTLA4 and PD-1/PD-L1 axis (2–5)], monospecific (6) and bispecific (7) monoclonal antibodies, immune-stimulatory agents such as BCG (8), cancer vaccines (9), and the adoptive transfer of tumor-reactive immune cells (10).

Adoptive cell therapy (ACT) is a personalized strategy that involves infusion of *ex vivo*-expanded endogenous (pre-existing) tumor-reactive T cell repertoires, such as tumor-infiltrating lymphocytes (TILs) (11), and endogenous T cell therapy (ETC) (12), or the generation of artificial tumor-reactive T cells (13), such as engineered T cells expressing transgenic T cell receptors (TCR) or chimeric antigen receptors (CAR) (14). While TILs are tumor-specific lymphocytes directly isolated from tumor resections, ETC are tumor-reactive T cells isolated from the peripheral blood of patients (15). Engineered TCR- and CAR-T cells are leukapheresis blood-derived T cells genetically modified *ex vivo* in order to specifically recognize a tumor-associated antigen (TAA) *via* mRNA electroporation (16), lentiviral (17) or retroviral (18) transduction, transposon mediated modification (19) or *via* CRISPR/Cas9 gene editing (20).

### Tumor-Infiltrating Lymphocytes

TILs are T cells isolated from tumor fragments, *ex vivo*-expanded and reinfused back into pre-conditioned patients under a non-myeloablative lymphodepletion chemotherapy with high doses of interleukin-2 (IL-2) (21). TILs have shown impressive results in patients with metastatic melanoma (MM), where objective response rates of 40%–50% including complete tumor regression in 10%–20% of treated patients have consistently been reported by several independent centers (22–27). Although TILs can also be obtained from epithelial cancers (11, 28) such as breast (29, 30), ovarian (31), renal (32), gastrointestinal (33), pancreatic (34), cervical (35) or prostate (36) tumors, the reported response rates have been very modest (37).

In CAR-T-based ACT the major histocompatibility complex (MHC)-restricted peptide presentation is bypassed, but only a user-defined cell surface TAA can be recognized by the CAR. However, the use of CAR-T cells in solid tumors has been limited by organ toxicities related to activation of T cell effector functions through the CAR, since most TAAs are also found in normal tissues, raising the risk of on-target off-tumor toxicities (38). In contrast, TILs products are highly polyclonal; thus TIL-based ACT benefits from a multitarget T cell attack directed against multiple different and largely unknown antigens (39). Due to the complexity of identifying the antigen repertoire present in every tumor for which TIL detection has been reported it has been difficult to assess the specific antigens that are detected by TILs, but cloning studies have divided them into non-self and self-antigens, which can be further divided into another 3 major groups: cancer germline antigens (developmental proteins that are re-expressed in some cancerous but not adult/normal tissues), differentiation antigens (which can also be present in normal tissues but in a limited distribution) and foreign antigens (that arise from viral proteins in viral-associated cancers) (39). Nevertheless, several studies demonstrated that the effectiveness of TIL-based ACT in MM is based on the specific recognition of neoantigens (nonsynonymous somatic mutations) (40, 41).

Although TIL therapy is not yet approved by the FDA, several clinical trials are being performed (mainly for MM) (42), and current efforts for its approval are focused on optimizing the manufacturing process (43), and its application to other cancer types. In contrast to MM, the effectiveness of TIL-based ACT relies on the fact that the number of TILs isolated from other tumor types is lower and difficult to expand, as well as on the mutational burden and the characteristics of the tumor microenvironment (44). The “standard TIL” isolation protocol (“selected TILs”), is based on an initial pre-Rapid Expansion Protocol (preREP) stage which comprise the resection of fresh tumor specimens into small segments, their fragmentation and culture under high-dose IL-2 (6,000 IU/ml) conditions for 3–5 weeks (45). The outcome of this first expansion phase is rather variable, since the number of TILs present in the original tumor does not always correlates with the efficiency of the preREP process (46). Following this preREP step, individual TIL micro-cultures are assayed with IFN-γ ELISA/ELISPOT for the ability to recognize autologous tumor cells or HLA-A matched allogeneic melanoma cell lines (43). TIL micro-cultures displaying tumor reactivity against HLA-matched or autologous tumor cells are selected and expanded further in the REP stage under an allogeneic feeder co-culture with healthy donor irradiated (40 Gy) peripheral blood mononuclear cells (PBMC) in a 1:200 ratio with 30 ng/ml anti-CD3 (clone OKT3) and 6,000 IU/ml IL-2. After this REP stage, expanded TILs are transferred into culture bags, prepared and reinfused back into the patient. In addition to the manufacturing obstacles derived from TIL therapy, —it is an extremely personalized therapy which required specialized personal to manage pre- and REP stages as well as highly controlled conditions that guarantee their clinical use—, TIL expansion is a time-consuming protocol that in some cases is not viable due to the rapid clinical deterioration of some melanoma patients from which those TIL were initially isolated. Due to the limitations of the selected TIL method, a modified TIL production protocol was developed and tested in clinical trials both at the Surgery Branch, NCI and the Sheba Medical Center, Israel (47–49). With this modified method, named the “young TIL” protocol, all TIL micro-cultures generated from individual fragments are pooled together as one single bulk TIL culture, eliminating the tumor-reactivity assay (50).

Along with other limitations that TIL-based ACT implies, such as their reduced proliferative capacity and *in vivo* persistence after the reinfusion to the patient, TILs are in essence highly differentiated effector cells (39), which differentiate from a T effector phenotype to late-stage effector memory cells (51–53). Current efforts also implies selection of tumor-reactive TILs with the co-stimulatory marker 4-1BB/CD137 (34, 54–59) and alternatively, with PD-1/CD279 (60–62), although these approaches remain to be evaluated under clinical trials (63).

The success of TIL-based ACT in MM is based mainly in the high mutational burden and neoantigen emergence rates and in the sustained antitumor reactivity exhibited by this type of cancer (42). However, these characteristic are usually absent in most epithelial tumors such as those mentioned above. In contrast to MM, other solid tumors from which TIL isolation and production have been achieved lack a high mutational load or neoantigen burden and exhibit a scarce antitumor reactivity, which combined to the heterogeneous CD4^+^ or CD8^+^ lymphocyte or innate-like and myeloid infiltrates, and the wide variety of metastases types, are currently hindering the applicability of TIL-based ACT (64). In this context, genetically-modified TILs, named “*synthetic* TILs”, could emerge as an effective ACT beyond MM.

### Synthetic TILs in Cancer Therapy

Given the therapeutic potential of TIL-based ACT, almost in parallel with de development of TIL protocols, the genetic modification of TILs has been explored in order to improve their tumor-homing ability after *ex vivo* expansion and reinfusion into patients (**Table 1**). In early 90’, feasibility of TIL gene transfer after transduction with the retroviral vector N2 encoding the bacterial gene for neomycin-resistance (NeoR) was analyzed, concluding that those NeoR-TILs could be used for studying TIL trafficking and survival *in vivo* with no growth detriment or cytokine mRNA pattern alterations (65). Based on these results, the same group studied the safety of reinfusion of genetically modified NeoR-TILs in five MM patients. No adverse effects were reported, PCR analysis detected NeoR-TILs in the circulation after three weeks and in the tumor deposits after 64 days (66, 80), and long-term viable NeoR-TILs after cell infusion were observed (67).

**Table 1:** *Synthetic* tumor-infiltrating lymphocytes (TILs) in cancer therapy.

Objective	Gene Modification	Vector/Genetic Technology	TumorType	# Patients	Reference
TIL trafficking	*NeoR*	RV	M	6	(65)
	*NeoR*	RV	MM	5	(66)
	*NeoR*	RV	MM MRCC	3 2	(67)
	*NeoR*	RV	EOC	ND	(68)
Improvement of TIL cytotoxicity	*TNFα*	RV	ND	15	(69)
	*TNFα* *^a^*)	RV	MM	11	(70)
	*TRAIL+IL2*	MEV	RCC	10	(71)
	*IL2*	RV	MM	13	(72)*
	*NFAT. IL-12*	RV	MM	33	(73)*
Enhancement of TIL homing toward tumor sites	*CXCR2*	RV	M	10	(74)
	*CXCR2*	LV	MM	ND	(75)
	*CXCR2*	RV	MM	10	(76, 77)*
	*CXCR1*	BEV	MM	40	(78)
Prevention of TIL exhaustion	*PD-1*	ZFN-mediated gene editing	MM	3	(79)

a Additional treatment with trans-retinoic acid. BEV, Bacterial Expression Vector; EOC, Epithelial Ovarian Carcinoma; LV, Lentiviral Vector; MEV, Mammalian Expression Vector; M, Melanoma; MM, Metastatic Melanoma; MRCC, Metastatic Renal Cell Carcinoma; ND, Not Determined; RV, Retroviral Vector; *Clinical Trial (https://clinicaltrials.gov).

Summary of the different strategies undergone to date for the generation of genetically modified TILs.

However, not only MM-derived TILs have been explored for gene modification. Transduced CD4^+^ and CD8^+^ TILs with the G1Na retroviral vector (encoding for NeoR) have been used to study the *in vivo* trafficking of ovarian-derived TILs (68).

## Synthetic TILs With Increased Cytotoxic Potential

Other approaches have explored the genetic engineering of TILs with secreted proteins such as tumor necrosis factor (TNF). Hwu et al. (69) demonstrated that TILs could be retrovirally transduced with TNF-α, and although the secreted levels *in vitro* were lower than expected, these levels could be increased by replacing the transmembrane region of TNF with the IFN-γ signal peptide or after treatment with retinoic acid (70).

TNF-related apoptosis-inducing ligand (TRAIL) and IL-2 have also being transfected into TILs isolated from renal cell carcinoma (RCC) resulting in improved cytotoxicity activity (71). A clinical trial explored whether retrovirally transduced IL-2 secreting MM-derived TILs could enhance their *in vivo* survival after adoptive transfer. IL2-secreting *synthetic* TILs improved their *in vitro* survival in the absence of added IL-2, but the *in vivo* survival or clinical were not enhanced (72). The potential of IL-12 as a putative enhancer of antitumor activity has been studied in TILs isolated from MM patients. Based on previous studies showing tumor cell toxicity associated with constitutive IL-12 secretion (81), Zhang et al. (73 *)* developed a system in which a single-chain human IL-12 driven by a nuclear factor of activated T cells (NFAT) inducible promoter was selectively secreted at the tumor site after the TCR engagement. However, although objective responses were observed, the clinical toxicities likely associated with IL-12 secreted by *synthetic* NFAT.IL-12-TILs makes it imperative to improve the approach before undertaking further studies.

## Synthetic TILs With Enhanced Tumor Homing Ability

**Figure 1:**
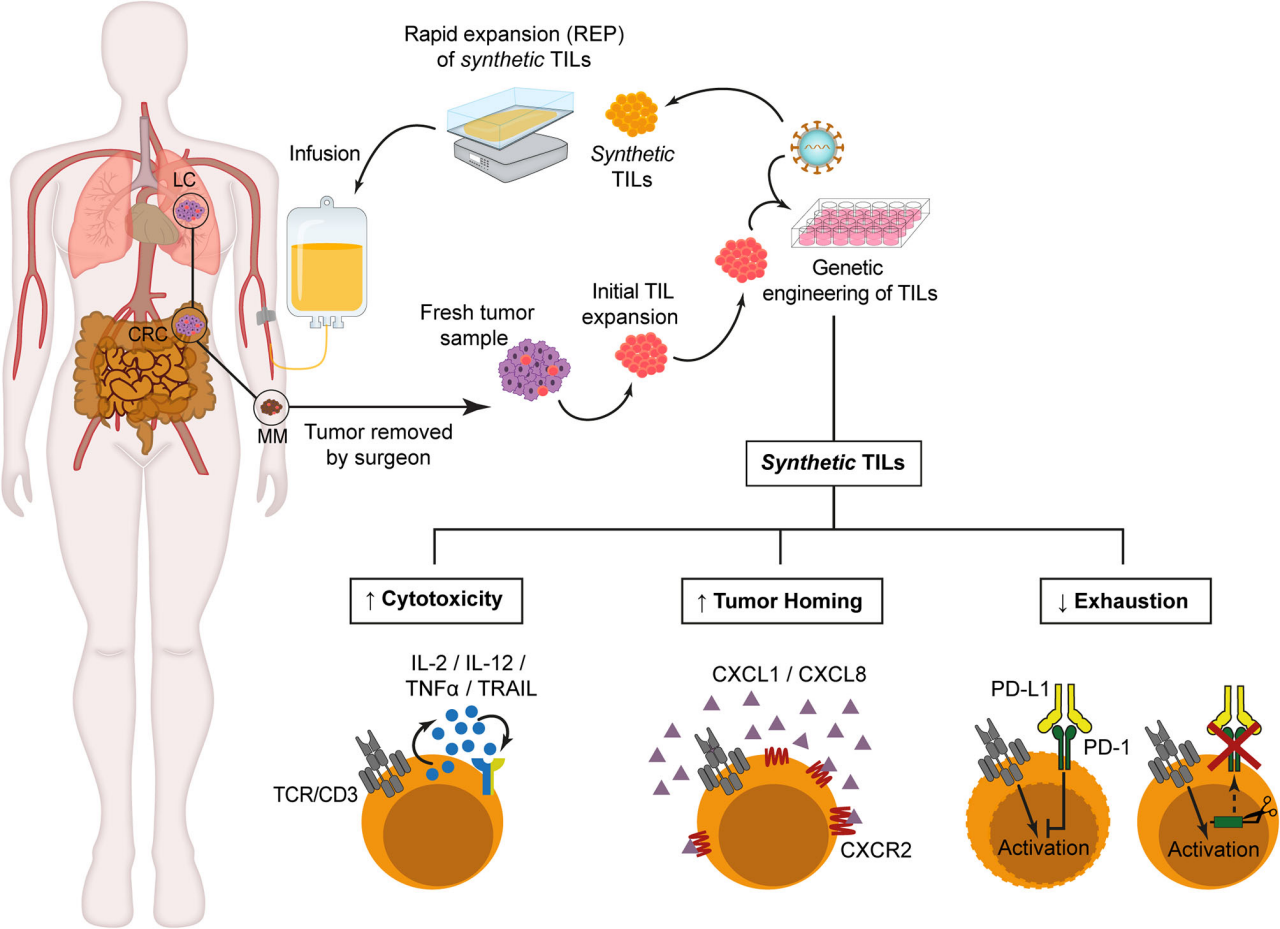
*Synthetic* TILs manufacturing. After initial TILs isolation from fresh tumor fragments, retroviral or lentiviral vectors are used to genetically modified TILs in order to improve cytotoxicity, enhance tumor homing or reduce T cell exhaustion. Then, *synthetic* TILs are ex vivo expanded for 14 days in the presence of allogeneic irradiated peripheral blood mononuclear cells, soluble anti-CD3 antibodies and IL-2. Prior to synthetic TIL infusion, the patients receive lymphodepleting chemotherapy to ensure TIL persistence and expansion. CRC, Colorectal Cancer. LC, Lung Cancer. MM, Metastatic Melanoma..

Once proved that TILs can be efficiently engineered using different strategies, recent studies have been focusing on improving TIL migration toward tumor sites after re-infusion (**Figure 1**). With this aim, several groups have explored the generation of *synthetic* TILs expressing chemokine receptors for different chemokines secreted by tumor cells, such as CXCR2, which is the receptor for several chemokines such as CXCL1 and CXCL8. Initial studies demonstrated that recombinant as well as tumor cell line-derived CXCL1 induced chemoattraction *in vitro* and IFN-γ secretion of CXCR2-engineered T cells (74). *In vivo* studies in two xenograft tumor models have also shown that melanoma antigen-specific CXCR2-engineered T cells improved tumor migration and antitumor activity in mice bearing MC38/gp100 tumors or CXCL1-expressing tumors (82). These findings have been validated in NOG mice bearing subcutaneous human melanoma xenografts, in which increased tumor homing and infiltration by CXCR2-engineered T cells was observed (75). In addition, a clinical trial with CXCR2-engineered in MM patients is currently ongoing (76). The methodology developed for this clinical trial has been described by Forget et al. (77) and includes retroviral transduction and TIL expansion (83). Another chemokine studied as a possible target for *synthetic* TIL generation is CXCR1, which in contrast to CXCR2, is expressed at low levels in MM-derived TILs. Sapoznik et al. (78) demonstrated that CXCR1-engineered TILs migrated *in vitro* more efficiently toward melanoma or recombinant CXCL8 without altering effector function of migrating TILs.

## Reducing T Cell Exhaustion With Synthetic TILs

One of the critical challenges that must be solved for clinical approval of TIL-based ACT concerns the durability of TIL responses (**Figure 1**). PD-1 ligands, PD-L1/L2 are expressed in several tumor types, and the interaction with its receptor triggers effector T cell function inhibition and T cell exhaustion, developing a suppressive microenvironment which prevents antitumor activity. Rosenberg’s group had previously described that isolated tumor-reactive TILs from MM patients expressed PD-1 (61, 84), so they analyzed whether a permanent inhibition of PD-1 in engineered-TILs through zinc finger endonucleases (ZFN) gene-editing technique could improve the effectiveness of TIL after infusion. Beane et al. (79) demonstrate a significant decrease in the number of tumors after treatment with ZFN-PD-1 KO TILs as well as an improved *in vitro* effector function through TNF-α, GM-CSF and IFN-γ secretion after co-culture with tumor cells, with a predominant effector memory-like phenotype in engineered TILs, and no detectable proliferative defects or tumor formation in NSG mice (79). Nevertheless, safety and efficiency for clinical treatment remains to be tested.

## Conclusions and Future Perspectives

TIL-based ACT in combination with high-dose IL-2 have been shown to be an effective clinical strategy in MM patients, and to a lesser extent, in other tumors. Although many issues remain to be addressed, especially regarding the relatively long generation process and the requirement for GMP facilities and trained personnel, the early-treatment costs are considerably lower than those of anti-CTLA4 mAb in MM (85). Given than TILs are naturally infiltrating cells, they can also serve as biomarker to predict the clinical efficacy of immunotherapies enhancing antitumor adaptive responses (86) Interestingly, different approaches have recently demonstrated that during the *ex vivo* process necessary for TIL generation, these tumor-specific T cells can be efficiently genetically modified in order to enhance their cytotoxicity, tumor homing or to reduce T cell exhaustion. The resulting cellular product, called *synthetic* TILs in this review, is at the very beginning of their evolution and could eventually transform the current immunotherapy landscape. By using different genetic engineering strategies and/or gene editing systems, we can speculate that it will be possible to generate personalized *synthetic* TIL-based ACTs addressing the particular tumor characteristics, with the aim of counteracting the specific tumor evasion mechanisms that are operative in a given patient, or redirecting other immune cells against the tumor. Furthermore, the identification of the TIL mutanome, the specific mutated neoantigens recognize by TILs (86), will provide rationale to develop “à la carte” neoantigen-specific *synthetic* TILs, or combinations thereof that could be significantly more effective than populations of potentially tumor-reactive TILs obtained by conventional enrichment protocols.

## Author Contributions

AJ-R and LA-V contributed to the conception of the work and wrote the manuscript. AJ-R and CD-A conceptualized and performed the figure and DN-A developed the table. All authors contributed to the article and approved the submitted version.

## Funding

AJ-R was supported by CRIS Cancer Foundation. CD-A was supported by the Spanish Ministry of Economy and Competitiveness (PRE2018-083445). LA-V was supported by grants from the Spanish Ministry of Economy and Competitiveness (SAF2017-89437-P, RTC-2017-5944-1), the CRIS Cancer Foundation (FCRIS-IFI-2018), and the Spanish Association Against Cancer (AECC, 19084).

## Conflict of Interest

The authors declare that the research was conducted in the absence of any commercial or financial relationships that could be construed as a potential conflict of interest.

## Abbreviations

ACT, adoptive cell therapy; CAR, chimeric antigen receptor; ETC, endogenous T cell therapy; IL-2, inteleukin-2; mAb, monoclonal antibody; MHC, major histocompatibility complex; MM, metastatic melanoma; NeoR, bacterial gene for neomycin-resistance; NFAT, nuclear factor of activated T cells; PBMC, peripheral blood mononuclear cells; preREP, pre-Rapid Expansion Protocol; RCC, renal cell carcinoma; REP, rapid expansion protocol; TAA, tumor-associated antigen; TIL, tumor-infiltrating lymphocyte; TCR, T cell receptor; TNF, tumor necrosis factor; TRAIL, TNF-related apoptosis-inducing ligand; ZFN, zinc finger endonucleases.
